# 1, 742例Ⅳ期非小细胞肺癌的预后分析

**DOI:** 10.3779/j.issn.1009-3419.2011.04.11

**Published:** 2011-04-20

**Authors:** 红 彭, 美丽 马, 宝惠 韩

**Affiliations:** 200030 上海，上海市胸科医院门诊办公室 Outpatient Ofce, Shanghai Chest Hospital, Shanghai 200030, China

**Keywords:** 肺肿瘤, 转移, 预后, Lung neoplasms, Metastasis, Prognosis

## Abstract

**背景与目的:**

目前非小细胞肺癌（non-small cell lung cancer, NSCLC）仍是导致癌症死亡的首要原因，本研究旨在探讨影响晚期NSCLC预后的重要因素。

**方法:**

收集2000年1月4日-2008年12月25日1, 742例Ⅳ期NSCLC临床资料，所有病例经细胞学或组织病理学确诊。分析性别、年龄、吸烟史、病理类型、分型、临床TN分期、转移器官数目、治疗方法对预后的影响，应用*Kaplan-Meire*方法计算生存率，*Log-rank*检验生存率差别，采用*Cox*多因素回归对预后因素进行分析。

**结果:**

本组1, 742例患者的中位生存期为10.0个月（9.5个月-10.5个月），1年、2年、3年、4年、5年生存率分别为44%、22%、13%、9%、6%。单器官发生转移与多器官转移中位生存期分别为11个月*vs* 7个月（*P* < 0.001）。不同器官发生转移后生存期不同，中位生存期分别为肺12个月（11.0个月-12.9个月），骨9个月（8.3个月-9.6个月），脑8个月（6.8个月-9.1个月），肝、肾上腺、远处淋巴结转移均为5个月（3.8个月-6.1个月），皮下3个月（1.7个月-4.3个月）。腺癌患者1, 086例（62%），鳞癌305例（17.5%），中位生存期分别为12个月*vs* 8个月（*P* < 0.001）。化疗与最佳支持治疗者中位生存期分别为11个月*vs* 6个月（*P* < 0.001）。放疗与否的中位生存期分别为11个月*vs* 9个月（*P*=0.017）。

**结论:**

性别、年龄、大体分型、病理类型、临床T分期、N分期、转移器官数目、吸烟史、治疗方法是晚期NSCLC预后的独立影响因素。

## Abstract (en)

**Background and objective:**

At present non-small cell lung cancer (NSCLC) is still the leading cause of death induced by cancer. The aim of this study is to investigate the prognostic factors of advanced NSCLC.

**Methods:**

Total 1, 742 cases of stage Ⅳ NSCLC data from Jan 4, 2000 to Dec 25, 2008 in Shanghai Chest Hospital were collected, confrmed by pathological examinations. Analysis was made to observe the impact of treatment on prognosis in gender, age, smoking history, pathology, classifcation, clinical TNM stage. Survival rate, survival difference were evaluated by *Kaplan-Meire* method and *Logrank* test respectively. The prognosis were analyzed by *Cox* multivariate regression.

**Results:**

The median survival time of 1, 742 patients was 10.0 months (9.5 months-10.5 months). One, two, three, four, and fve-year survival rates were 44%, 22%, 13%, 9%, 6% respectively. The median survivals of single or multiple metastasis were 11 months *vs* 7 months (*P* < 0.001). Survival time were different in metastasic organs, with the median survival time as follows: lung for about 12 months (11.0 months-12.9 months), bone for 9 months (8.3 months-9.6 months), brain for 8 months (6.8 months-9.1 months), liver, adrenal gland, distannt lymph node metastasis for 5 months (3.8 months-6.1 months), and subcutaneous for 3 months (1.7 months-4.3 months). The median survival times of adenocarcinoma (*n*=1, 086, 62%) and squamous cell carcinoma cases (*n*=305, 17.5%) were 12 months *vs* 8 months (*P* < 0.001). The median survival time of chemotherapy and best supportive care were 11 months *vs* 6 months (*P* < 0.001); the median survival times of with and without radiotherapy were 11 months *vs* 9 months (*P*=0.017).

**Conclusion:**

Gender, age, gross type, pathological type, clinical T stage, N stage, numbers of metastatic organ, smoking history, treatment of advanced non-small cell lung cancer were independent prognostic factors.

全世界范围内每年有超过100万的患者被诊断为肺癌，其中80%为非小细胞肺癌（non-small cell lung cancer, NSCLC）。目前NSCLC仍是导致癌症死亡的首要原因^[1]^。虽然手术是肺癌的首选治疗手段，但75%以上的肺癌患者在确诊时已为不可手术切除的Ⅲb期或Ⅳ期。近年来，由于有计划、合理地综合应用现有的几种治疗手段，晚期肺癌的生存有所延长。现回顾性分析2000年1月4日-2008年12月25日在我院治疗的1, 742例Ⅳ期NSCLC的预后因素，以便为临床治疗提供可参考的数据。

## 1 材料与方法

### 1.1 临床资料

2000年1月4日-2008年12月25日在上海交通大学附属上海市胸科医院住院、经电话随访的1, 742例Ⅳ期NSCLC患者。所有患者通过气管镜、肺穿刺、淋巴结穿刺、痰检、纵隔镜、胸腔镜、胸水细胞学检查等明确病理类型，同时胸部CT、腹部B超及上腹部CT、骨扫描、头颅CT或MRI检查或全身PET检查明确重要器官包括肺、骨、脑、肝、肾上腺有无转移。B超、PET或穿刺明确有无远处淋巴结转移，穿刺明确有无皮下转移。所有患者均符合以下诊断标准：①经细胞学或组织病理学检查证实为NSCLC；②根据1997年国际抗癌联盟公布的修订后的肺癌国际分期，肿瘤分期为Ⅳ期；至随访结束时，死亡1, 527例，存活215例，相关临床特征见表 1。全组男性1, 132例，女性610例，男:女为1.86。年龄19岁-87岁，平均年龄为61.5岁。多器官转移（发生转移器官数目 > 1个）496例，单器官转移（转移器官数目=1）1, 246例，其中骨转移962例，肺转移752例，脑转移252例，肝转移165例，肾上腺转移79例，皮下转移29例，远处淋巴结转移30例。治疗方式包括化疗、放疗、靶向治疗、手术、最佳支持治疗等。未化疗者491例，化疗1, 251例；放疗422例；手术172例（其中包括同侧两叶肺切除或全肺切除或肺叶切除加楔形切除术83例，原发肺肿瘤伴肋骨转移2例，肺肿瘤伴脑转移瘤4例，剖胸探查、胸腔镜、纵隔镜探查32例，姑息性手术51例），靶向治疗患者多为门诊就诊，资料不全，故未纳入统计中。

**1:** 晚期NSCLC预后影响因素的单因素分析结果 Advanced NSCLC prognostic factors in univariate analysis

Clinical factor	*n* (%)	Median survival time (month)	*P*
Gender			< 0.001
Male	1, 132 (64.9)	9	
Female	610 (35.1)	13	
Age (year)			0.110
< 65	969 (55.6)	10	
≥ 65	773 (44.4)	9	
Smoking history			< 0.001
No	847 (48.6)	13	
Yes	895(51.4)	8	
Gross type			< 0.001
Central	778 (44.7)	8	
Peripheral	964 (55.3)	11	
Pathology			< 0.001
Adenocarcinoma	1, 086 (62.3)	12	
Squamous cell carcinoma	305 (17.5)	8	
Adenosquamous	68 (3.9)	8	
Indefinite	280 (16.1)	7	
Large cell carcinoma	3 (0.2)	14	
T stage			< 0.001
1	59 (3.4)	15	
2	847 (48.6)	10	
3	159(9.1)	8	
4	643 (36.9)	10	
X	34(2.0)		
N stage			< 0.001
0	136 (7.8)	14	
1	168 (9.6)	13	
2	782 (44.9)	10	
3	568 (32.6)	9	
X	88 (5.0)		
Number of organs with metastasis			< 0.001
1	1, 246 (71.5)	11	
> 1	496 (28.5)	7	
Chemotherapy			< 0.001
No	491 (28.2)	6	
Yes	1, 251 (71.8)	11	
Cycle			< 0.001
0	491 (28.2)	6	
≤ 4	882 (50.6)	9	
> 4	369(21.2)	23	
Radiotherapy			0.017
Yes	422 (24.2)	11	
No	1, 320 (75.8)	9	
X: unknown.			

### 1.2 方法

回顾性分析1, 742例患者病历资料，随访方式为电话随访，末次随访时间为2009年6月30日。本研究评估的主要研究终点为总生存期（overall survival, OS），即从确诊日期开始至患者死亡或末次随访时间，以月为单位。次要研究终点为1年、2年、3年、4年、5年生存率。

### 1.3 统计学方法

应用SPSS 13.0统计软件进行数据分析。单因素分析采用*Kaplan-Meire*方法和*Log-rank*检验，多因素分析采用*Cox*回归模型。*P* < 0.05为差异有统计学意义。

## 2 结果

### 2.1 生存情况

本组1, 742例患者中位生存期为10.0个月（9.5个月-10.5个月），1年、2年、3年、4年、5年生存率分别为44%、22%、13%、9%、6%，生存曲线见图 1。本组晚期NSCLC常见转移部位依次为骨（962例）、肺（752例）、脑（252例）、肝（165例）、肾上腺（79例）、远处淋巴结（30例）、皮下转移（29例），发生率分别为55%、43%、14%、8%、4.5%、1.6%、1.6%。单器官转移中位生存期11个月（10.3个月-11.7个月），多器官转移中位生存期7个月（6.1个月-7.8个月）。不同器官发生转移后生存期不同，中位生存期分别为肺12个月（11.0个月-12.9个月），骨9个月（8.3个月-9.6个月），脑8个月（6.8个月-9.1个月），肝、肾上腺、远处淋巴结转移均为5个月（3.8个月-6.1个月），皮下3个月（1.7个月-4.3个月）。

**1:**
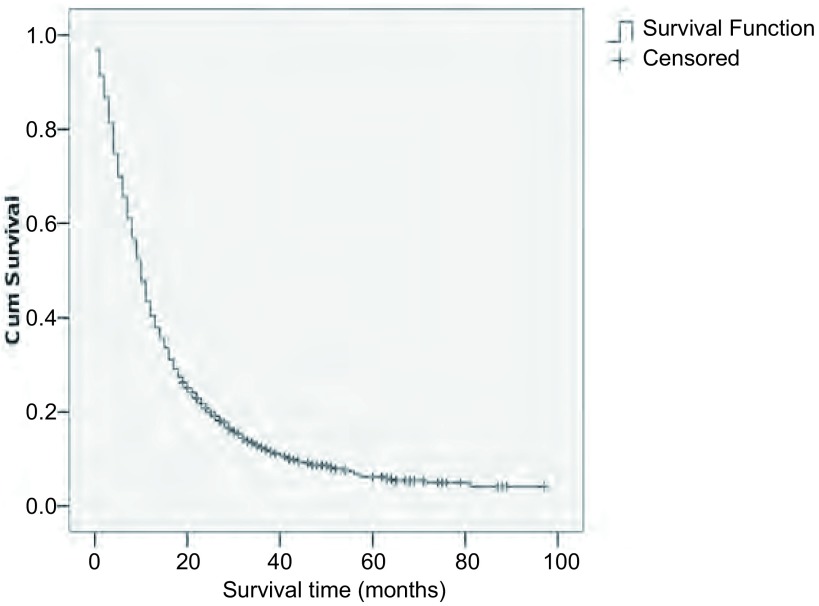
1, 742例Ⅳ期NSCLC的生存曲线 1, 742 cases of Ⅳ NSCLC survival curve

### 2.2 预后因素分析

#### 2.2.1 单因素分析

对性别、年龄、吸烟史、病理类型、临床T分期、N分期、转移器官数目、治疗方法进行单因素分析结果显示如表 1。腺癌患者1, 086例（62%），鳞癌305例（17.5%），中位生存期分别为12个月*vs* 8个月，差异有统计学意义（*P* < 0.001）。化疗与最佳支持治疗者中位生存期分别为11个月*vs* 6个月，差异有统计学意义（*P* < 0.001）。放疗与否的中位生存期分别为11个月*vs* 9个月，差异有统计学意义（*P*=0.017）。

单因素分析显示患者性别、年龄、吸烟史、病理类型、大体分型、临床T分期、N分期、转移器官数目、治疗方法均与预后有关。其中女性、 < 65岁、无吸烟史、周围型、腺癌、单器官转移、TN分期较早且接受放化疗治疗者预后较好。

#### 2.2.2 多因素分析

将单因素分析有统计学意义的影响因素引入*Cox*回归模型进行多因素分析，结果显示性别、年龄、大体分型、病理类型、临床T分期、N分期、转移器官数目、吸烟史、治疗方法是晚期NSCLC预后的独立影响因素（表 2）。

**2:** 晚期NSCLC预后影响因素的*COX*多因素回归分析结果 Advanced NSCLC prognostic factors in multivariate regression analysis of *COX*

Variable	B	SE	Wald	df	*P*	OR
Gender	0.354	0.078	20.333	1	< 0.001	1.424
Age	-0.173	0.058	8.927	1	0.003	0.841
Pathology type	0.193	0.055	12.186	1	< 0.001	1.213
Pathology			19.084	4	0.001	
Adenocarcinoma	-0.223	0.093	5.765	1	0.016	0.800
Squamous	-0.278	0.077	12.989	1	< 0.001	0.757
Indefinite	0.078	0.148	0.276	1	0.599	1.081
Adenosquamous	-0.780	0.719	1.177	1	0.278	0.458
Anatomy type	-0.359	0.092	15.091	1	< 0.001	0.699
T stage	0.064	0.027	5.479	1	0.019	1.066
N stage	0.194	0.033	34.829	1	< 0.001	1.214
Number of organs with metastasis	0.402	0.060	44.668	1	< 0.001	1.495
Smoking history	0.164	0.074	4.842	1	0.028	1.178
Chemotherapy	0.626	0.109	33.248	1	< 0.001	1.870
Cycle	-0.974	0.074	173.162	1	< 0.001	0.377

## 3 讨论

肺癌是目前发病率和死亡率增长最快、对人类健康和生命威胁最大的恶性肿瘤之一。肺癌预后差，长期生存率较低。本组1, 742例患者中位生存期为10.0个月（9.5个月-10.5个月），1年、2年、3年、4年、5年生存率分别为44%、22%、13%、9%、6%。本文重点对*Cox*多因素分析有意义的独立变量进行讨论。

### 3.1 年龄

肺癌好发年龄为60岁-65岁，约50%病例 > 65岁，30%病例 > 70岁。本组肺癌患者平均年龄为61.5岁。本研究显示 < 65岁肺癌预后优于≥65岁患者，分析原因可能与老年人多数伴有不同程度的心脑血管病和潜在肝、肾功能储备力降低有关，另外老年人对外科手术治疗、放疗、化疗的耐受力较低^[2]^。但研究^[3]^显示对于行为状态良好、东部协作组体能状态评分（Eastern Cooperative Oncology Group Performance Status Scale, ECOG PS）0分-1分的老年患者，年龄不影响预后，故建议不要轻易放弃治疗。

### 3.2 性别和吸烟

本组77%男性吸烟，3.7%女性吸烟。不吸烟者预后好于吸烟者，女性预后好于男性。吸烟对心肺功能均有非常不利的影响，而且不吸烟NSCLC原发病灶多位于肺外周，咯血风险低，少或无伴发疾病。故不吸烟者预后较吸烟者好。另外不吸烟和吸烟NSCLC患者表皮生长因子受体（epidermal growth factor receptor, EGFR）突变率分别为45%和7%，且EGFR突变率与吸烟数量呈负相关。EGFR激活型突变与表皮生长因子受体酪氨酸激酶抑制剂（epidermal growth factor receptor tyrosine kinase inhibitor, EGFR-TKI）疗效明显相关，是选择进行EGFRTKI（如吉非替尼或厄洛替尼）治疗和预测疗效的重要指标^[4]^。肺腺癌患者中*K-ras*的突变率为15%-30%.并且与吸烟相关。*K-ras*突变与原发性EGFR-TKI耐药相关^[5]^。随着EGFR-TKI等靶向治疗药物的出现，使*EGFR*突变者获益，从而使女性、不吸烟者预后好转。然而靶向治疗患者后续多为门诊就诊，随访较困难，本研究中靶向治疗资料不全，故暂未纳入统计中。

### 3.3 病理类型

本研究显示腺癌预后好于非腺癌。分析原因可能与鳞癌患者多以中央型为主，易出血，病灶增大后易引起肺不张、阻塞性肺炎等并发症多；对放化疗、靶向治疗的敏感性较腺癌差有关；另外鳞癌患者多有较长吸烟史，心肺功能受吸烟影响而减弱。对于腺鳞混合型患者，化疗药物不能同时有效抑制两种不同细胞，疗效较单纯细胞类型癌差。另外治疗方面，Ⅲ期临床研究ECOG^[6]^证实贝伐珠单抗可较为安全地为非鳞癌患者带来生存获益；研究^[7]^显示培美曲塞均对非鳞癌患者表现出更好的疗效，且腺癌较鳞癌在治疗上更有优势。

### 3.4 TNM分期

第7版新TNM分期，内容包括：T1分为T1a（≤2 cm）、T1b（> 2 cm, ≤3 cm）；T2分为T2a（> 3 cm, ≤5 cm）、T2b（> 5 cm, ≤7 cm）；肿瘤 > 7 cm由原来的T2归为T3。原发肿瘤同一肺叶出现其它癌结节由原来的T4归为T3；原发肿瘤同侧胸腔内不同肺叶出现癌结节由原来的M1归为T4；胸膜播散（恶性胸腔积液、心包积液或胸膜结节）归为M1^[8]^。将原发肿瘤同侧胸腔内不同肺叶出现癌结节由原来的M1归为T4，并将T4N0-1M0由Ⅲb期改为Ⅲa期，提示老分期标准中部分患者可以纳入手术范围，表明这类患者较以往认为有更好的预后，不应放弃手术治疗。本回顾性研究的分期是采用第5、6版分期，研究结果显示TN分期是预后的独立影响因素，从而验证了老版本分期标准的科学性。值得一提的是本研究中172例手术患者中有83例（同侧两叶肺切除或全肺切除或肺叶切除加楔形切除术）患者根据第5、6版分期为M1（同侧胸腔内不同肺叶出现癌结节）患者，而根据第7版肺癌分期是可以手术。研究表明这些患者预后明显好于其他患者，中位生存期为18个月（15个月-21个月）。提示第7版肺癌分期标准能更准确地反映处于不同病期的患者预后，指导治疗。

### 3.5 治疗方式

#### 3.5.1 一线治疗

##### 3.5.1.1 化疗

与最佳支持治疗（best suppotive care, BSC）比较，以铂类为基础的化疗能够延长患者的生存期、提高生活质量。但含铂两药方案的疗效仍不令人满意，其总体有效率为25%-35%，中位疾病进展时间为4个月-6个月，中位OS为8个月-10个月，2年生存率低于20%^[9]^。美国国立综合癌症网络（National Comprehensive Cancer Network, NCCN）指南强调，化疗只能使行为状态良好（ECOG PS评分为0分-1分）者获益；对于老年或ECOG PS评分为2分的患者，单药化疗或含铂两药化疗方案均可以选择，应该根据患者的具体情况决定；全身化疗不能使ECOG PS评分为3分-4分者获益，所以不建议使用^[10]^。本研究显示化疗与否、化疗周期数是预后的独立因素。最佳支持治疗者的中位生存期为6个月，化疗组为11个月（*P*=0.001）。化疗周期≤4组、 > 4组的中位生存期分别为9个月*vs* 23个月。

##### 3.5.1.2 EGFR-TKI治疗

OCHS等^[11]^的EAP（expanded access pmgram)临床研究分层分析显示女性、东方人/亚洲人种、不吸烟者、肺腺癌的中位生存期较长。Nokihara等^[12]^研究结果表明对于未接受过化疗的患者，吉非替尼与含铂化疗方案的疗效相近，而后的交叉用药显示，先用吉非替尼并未对后续的化疗产生不良影响。故对伴有EGFR突变者，可以推荐一线使用吉非替尼。对于EGFR突变阴性或突变不明者，一线治疗首选化疗。

##### 3.5.1.3 抗VEGF单抗治疗

ECOG 4599研究^[13]^结果显示，在卡铂联合紫杉醇方案的基础上加用贝伐珠单抗（人源化抗VEGF单抗）一线治疗Ⅲb期和Ⅳ期NSCLC，与单纯卡铂和紫杉醇方案化疗比较，能够提高有效率（27% *vs* 10%, *P* < 0.000, 1）、延长中位生存期（6.4个月*vs* 4.5个月，*P* < 0.000, 1）和OS（12.5个月*vs* 10.2个月，*P*=0.007, 5）。治疗组的中位总生存期超过1年，提示抗血管生成治疗具有重要作用。

#### 3.5.2 二/三线治疗

患者在一线治疗过程中或结束后病情进展，可以应用多西紫杉醇、培美曲塞或厄洛替尼单药作为二线治疗。三线治疗与安慰剂比较，厄洛替尼能够使患者获益^[13]^。
